# Carbapenem-Resistant *Pseudomonas aeruginosa*–Carrying VIM-2 Metallo-β-Lactamase Determinants, Croatia

**DOI:** 10.3201/eid0908.020373

**Published:** 2003-08

**Authors:** Sanda Sardelic, Lucia Pallecchi, Volga Punda-Polic, Gian Maria Rossolini

**Affiliations:** * University Hospital and School of Medicine Split, Split, Croatia; † University of Siena, Siena, Italy

**To the Editor**: Carbapenem-hydrolyzing enzymes of the VIM-type (six different variants are known: VIM-1, VIM-2, VIM-3, VIM-4, VIM-5, and VIM-6) are new molecular class B metallo-β-lactamases. These enzymes have recently been identified in carbapenem-resistant isolates of *Pseudomonas aeruginosa* and other gram-negative nonfermenters from European countries in the Mediterranean basin (Italy, France, Greece, Spain, Portugal, and Turkey), as well as in Far East countries *(*Korea, Taiwan, and Singapore) and the United States (*1**–**3*, Midilli et al., GenBank accession no. AY144612, Koh et al., GenBankaccession no. AY165025). Similar to *bla*_IMP_, *bla*_VIM_ genes are located on mobile gene cassettes inserted in the variable regions of integrons (*1*), a condition that provides a wide potential for expression and dissemination in gram-negative pathogens. VIM enzymes possess the broadest range of substrate hydrolysis and can degrade virtually all β-lactams, except monobactams (*4*).

According to a recent report, the overall resistance rate to imipenem in *P. aeruginosa* isolated from 17 representative laboratories in Croatia was 11% (range 0%–20%) (*5*). However, molecular basis of carbapenem resistance was not investigated.

In October 2000, two *P. aeruginosa* isolates with an unusual resistance profile were isolated from two Croatian patients (66 and 74 years of age, respectively) who underwent hysterectomies at the Split University Hospital. Both isolates were cultured from urine a week after surgery; a urinary catheter had been used for both patients who had become febrile and had signs and symptoms of urinary tract infection. Analysis of the macrorestriction profiles of chromosomal DNA of the two isolates by pulsed-field gel electrophoresis, carried out as described previously (*6*), indicated that the two isolates were clonally related (the two profiles were apparently identical). In routine antibiotic susceptibility testing, done by disk diffusion, both isolates showed a multidrug-resistant phenotype, including ureidopenicillins, piperacillin, piperacillin-tazobactam, ceftazidime, cefoperazone, cefepime, aztreonam, ciprofloxacin, gentamicin, netilmicin, imipenem, and meropenem.

MICs to imipenem and meropenem were high (>128 μg/mL). These findings suggested production of an acquired carbapenemase. In fact, crude extracts of the two isolates exhibited carbapenemase activity in a spectrophotometric assay (*7*) (imipenem hydrolyzing–specific activity was, in either case, >170 nmol/min/mg protein).

A colony blot hybridization, carried out as described with a *bla*_IMP_ and a *bla*_VIM_ probe (*6*), yielded a positive result with the latter probe. Polymerase chain reaction (PCR) amplification of the variable region of class 1 integrons, carried out as described previously by using primers designed on the 5′- and 3′-conserved segments of the integron (*8*), yielded a 4-kb amplification product from either isolate. Direct sequencing of these amplification products showed, in both cases, the presence of a *bla*_VIM-2_ allele located in a gene cassette inserted in the *attI* site of a class 1 integron.

The metallo-β-lactamase determinant was not transferred to *Escherichia coli* MKD135 or *P. aeruginosa* 10145/3 (*9*) in diparental mating experiments conducted on solid medium (the sensitivity of the assay was >1x10^-8^ transconjugants per donor). Plasmid extraction was performed with several techniques, including lysis with sodium dodecyl sulfate (*10*) and alkaline lysis conducted with a conventional method (*10*) or with the Nucleobond BAC100 system (Macherey-Nagel, Duren, Germany). Extraction of whole genomic DNA was also performed, as described (*8*). Plasmid DNA was not detected in any of these preparations, either when analyzed by agarose gel electrophoresis or after Southern blot hybridization analysis with a *bla*_VIM-2_ probe generated with PCR amplification of the entire *bla*_VIM-2_ gene. In Southern blots, a hybridization signal was only detectable in correspondence of the band of chromosomal DNA.

To our knowledge, this isolation is the first one of clinical strains producing acquired metallo-β-lactamase in Croatia. A similar finding underscores the progressive emergence of these determinants in different geographic areas and emphasizes the need for an early recognition of these strains. In fact, monitoring dissemination of new antibiotic resistance determinants is essential to enforce adequate control measures and adjust guidelines for antimicrobial chemotherapy in different hospital settings.
